# Perceived Effectiveness of Elder Abuse Interventions in Psychological Distress and the Design of Culturally Adapted Interventions: A Qualitative Study in the Chinese Community in Chicago

**DOI:** 10.1155/2013/845425

**Published:** 2013-12-25

**Authors:** XinQi Dong, E-Shien Chang, Esther Wong, Melissa Simon

**Affiliations:** ^1^Chinese Health, Aging, and Policy Program, Rush Institute for Healthy Aging, Rush University Medical Center, 1645 West Jackson Blvd, Suite 675, Chicago, IL 60612, USA; ^2^Chinese American Service League, Chicago, IL, USA; ^3^Northwestern University, Feinberg School of Medicine, Chicago, IL, USA

## Abstract

This qualitative study examines US Chinese older adults' views on the perceived effectiveness, challenges, and cultural adaptations of elder abuse interventions to psychological distress in the Chinese community in Chicago. A community-based participatory research approach was implemented to partner with the Chinese community. A total of 37 community-dwelling Chinese older adults (age 60+) participated in focus group discussions. Data analysis was based on grounded theory framework. Our findings suggest that older adults perceived social support, empowerment, and community-based interventions design as most effective to promote psychological well-being of victims. The perceived preferences were similar between elder abuse victims and non-victims. Strategies to culturally adapt evidence-based interventions were proposed with respect to nurturing filial piety values, familial integrations, and increased independence. Research and educational outreach initiatives were also discussed. This study has wide policy and practice implications for designing and deploying interventions to reduce psychological distress with respect to elder abuse outcome. Cultural relevancy of health interventions is important in the context of the Chinese communities. Collective federal, state, and community efforts are needed to support the culturally appropriate design and implementation of interventions suitable for the needs of the Chinese older adults.

## 1. Introduction

Elder abuse, sometimes referred to as elder mistreatment or elder maltreatment, is an important global health issue. Elder abuse manifests in various forms including physical abuse, sexual abuse, psychological abuse, caregiver neglect, and financial exploitation [1]. Available data suggest that 10% of US older adults aged 60 or older experience one or multiple forms of abuse [2, 3]. Despite the accessibility of Adult Protective Services in all fifty states, an overwhelming number of abused older adults may pass through healthcare system undiagnosed and overlooked. Associated with increased risk of morbidity and mortality, elder abuse continues to affect the quality of lives among vulnerable older adults [4–7]. Of particular importance is the adverse health outcome of elder abuse on victims' psychological well-being. Abused older adults exhibit symptoms of depression, anxiety, fear, and unworthiness, amongst other forms of psychological distress [8].

The associations between elder abuse and psychological distress highlight the urgent need for developing intervention programs addressing both elder abuse and psychopathology. Even though elder abuse has great public health relevance to health care professionals, social services providers, and the community gatekeepers and leaders, we have incomplete understanding of evidence-based prevention and intervention strategies to assist victims of elder abuse [9, 10]. A number of studies have started to examine the effectiveness of different intervention programs to reduce psychological distress, including increased social support for both victims and perpetrators [11–13], victim empowerment [14], advocacy interventions [14–16], and cognitive behavior activation [17]. However, research on interventions to reduce psychological distress with respect to elder abuse outcome remains scarce [18]. A recent systematic review further reported that existing interventions had limited effects on improving the social and psychological well-being of victims [10].

In addition, there exist structural, cultural, and social barriers in help seeking among the victims. For elder abuse victims, barriers to obtaining services may include physical frailty, cognitive impairment, and dependence on the abusers [19]. At the same time, victims may also serve in a caregiving or support capacity to their dependents or abusive family members, which in turn create different but equally challenging barriers to the victims' willingness and acceptance of needed services for themselves [20]. The issue of cultural diversity surrounding elder abuse further exemplifies a major complexity in advancing the field of elder abuse intervention. There exist knowledge gaps in understanding the cultural complexities of effective psychological well-being care supporting victims of elder abuse, particularly for minority older adults including African, Hispanic, and Asian populations [21].

Chinese population is one of the fastest growing minority groups in the U.S. Population estimates suggest that there are 4.0 million Chinese Americans in the United States, comprising 23% of the Asian American. Much of the health concern of Chinese older adults is deeply related to family caregiving guided by filial piety virtues. In Confucius teachings, filial piety dictates children's obligatory roles and responsibilities of caregiving to aging parents [22, 23]. Whereas respect for seniors is a deep-rooted cultural tradition, recent research suggests that elder abuse is an existing and pervasive health issue facing U.S. Chinese older adults [5, 24]. However, we are unaware of any studies that have examined the perceptions and preferred programs to reduce psychological distress from the perspectives of community-dwelling Chinese American older adults. Improved understanding of important factors shaping effective care for older adults' psychological well-being brings significant implications for health care professionals, social services providers, and health policy makers.

In order to expand our knowledge base on elder abuse interventions in a community-dwelling population of Chinese older adults, the goals of this qualitative study were to (1) elicit the perceived effectiveness of elder abuse interventions in reducing psychological distress, (2) explore similarities and differences in intervention preferences between the victim group and nonvictim group, and (3) identify strategies to culturally adapt evidence-based intervention programs to reduce psychological distress in the Chinese aging population.

## 2. Methods

### 2.1. Conceptual Framework and Definition

This study followed the conceptual framework of sociocultural context of elder abuse suggested by the National Research Council [1]. In this framework, elder abuse is defined as “intentional actions that cause harm or create a serious risk of harm (whether or not harm is intended) to a vulnerable elder by a caregiver or other person who stands in a trust relationship to the elder; or failure by a caregiver to satisfy the elder's basic needs or to protect the elder from harm.” The Socio-Cultural Context model focuses on the comprehensive assessment of vulnerability factors while considering the socio-cultural context and social embeddedness, which refers to the set of people in the social network between older adult and trusted others. It is the interactions among these components in which elder abuse may take place, while at the same time guiding the proposed analysis in this report.

### 2.2. Community-Based Participatory Research Approach

Community-based participatory research (CBPR) design has been reported as an important model for exploring the issues of elder abuse in minority communities [25, 26]. Described as “a systematic inquiry with the participation of those affected by the issue being studied, for the purpose of education and taking action or affecting social change” [27], CBPR allows researchers, community leaders, stakeholders, and members alike to form a synergistic collaboration for community changes. In order to fully engage Chinese community through the preparation, conduct, and findings dissemination of research processes, a community-academic collaboration was formed between Rush University Medical Center and Chinese American Service League (CASL), the oldest and largest social service agency dedicated to serving Chinese immigrants in the Midwest. In order to maximize cultural and linguistic sensitivity of our research efforts, we formed a community advisory board to provide overall guidance ranging from research conceptualization and preparation to the conduct and findings review [6]. Board members were stakeholders and leaders enlisted through civic, health, social and advocacy groups, community centers, and clinics. Residents and opinion leaders were also invited to join the bimonthly meetings.

Since the inception of this synergistic community-academic collaboration, elder abuse has been identified as a pervasive health issue facing the Chinese community. A number of initiatives were undertaken to improve our understandings in this complex public health concern. We investigated cultural perception of elder abuse in the Chinese community through focus group discussions and in-depth interviews [5–7]. A recent advocacy effort also included the commemoration of the World Elder Abuse Awareness Day (WEAAD). Proclaimed by President Obama, WEAAD is now observed every year on June 15 in the U.S. as the opportunity to learn the signs of elder abuse and to empower older adults with tools and information necessary to overcome abusive situations [28]. The Chicago community commemorated WEAAD for the first time in CASL. Residents were invited to join focus group discussions regarding their views on elder abuse interventions. All study procedures were approved by the Rush University Medical Center Institutional Review Board.

### 2.3. Study Design and Procedure

This study utilizes both survey questionnaires and semistructured focus group methods to investigate the experience of elder abuse among U.S. Chinese older adults. We invited seniors who experienced elder abuse as well as those who did not experience elder abuse to participate. Our sample size was 37. In order to ensure diverse opinions, we planned to recruit one-third of elder abuse victims and two-thirds of nonvictims in the study sample.

Elder abuse was measured using a self-reported instrument derived from the Hwalek-Sengstok Elder Abuse Screening Test (H-S/EAST) and the Vulnerability to Abuse Screening Scale (VASS) (Hwalek and Sengstock, 1986; Schofield and Mishra, 2003). Sample questions included if participants felt uncomfortable with someone in the family, have been called names or put down, or been forced by someone to do things their belongings without have been taken permission. In addition, basic sociodemographic information was collected, including age, sex, education, marital status, household, country of origin, years of residing in the U.S. and preferred language.

Participants were then invited to participate in semistructured focus group discussions. Health behavior and interventions are highly culturally mediated issues. Focus group technique is an important qualitative research technique. It is particularly well suited for an exploratory study for which previous health literature is limited and for generating hypotheses and models of human behavior [29]. Focus group participants were recruited in accordance with the following eligibility criteria: (1) aged sixty years or older, (2) self-identified as Chinese, and (3) reside in Chicago. Prior to survey questionnaires and focus group discussions, study participants gave written consents. All materials were prepared in simplified Chinese, traditional Chinese, and English. In order to ensure cultural sensitivity of the study, participants were then divided into four focus groups according to their preferred dialect, including Cantonese or Mandarin [30]. Focus group interviews were guided by trained facilitators affiliated with CASL. Participants' perception of elder abuse interventions, perceived barriers, and facilitators of intervention programs were explored by the following questions.

1. In general, what components in elder abuse interventions will be most effective to reduce psychological distress of abused seniors in our community?
2. What components or approach in elder abuse interventions will probably not work in reducing psychological distress of abused seniors in our community?
3. What are the creative ways or measures you can think of to improve the interventions components?
4. How do we overcome these challenges to promote intervention programs in accordance with Chinese cultural and linguistic contexts?

The length of discussions was determined by the levels of interaction among participants. The facilitators proceeded with topics when responses were exhausted.

### 2.4. Data Analysis

For analysis purpose, we performed descriptive statistical analysis based on survey results. In accord with the standard diagnosis of a positive screen, the screening was considered positive for elder abuse or neglect if a patient answered yes to any of the screening questions.

With respect to qualitative data, grounded theory provided a general framework to analyze and develop themes and theories [31–33]. A bilingual research assistant first transcribed audio recordings into Chinese transcripts (different dialects used the same Chinese characters). The original Chinese transcripts were then imported into NVivo software (NVivo, version 9) for analysis. Two bicultural and bilingual researchers analyzed data in Chinese iteratively. Following grounded theory, they first labeled the texts with key words and phrases. The key words were coded and analyzed for emerging categories. Two coders then compared and discussed their sets of categories collectively to evolve dominant themes. The categorization of each response was not finalized until consensus was reached. Each category was reviewed and a short summary was written for each category. Quotes from the Chinese transcripts that captured participants' opinions and sentiments were then translated into English and incorporated to support each theme. The essence of discussions could be truly captured by working with the original language in which the focus groups took place.

## 3. Results 

### 3.1. Characteristics of the Study Population by the Presence of Abuse

A total of 37 participants enrolled in the project (Table 1). Among these, 11 participants (29.7%) reported at least one item in the abuse screening tool in the domain of psychological, physical, financial abuse and exploitation. There were more men than women who screened positive for abuse. In the victim group, the majority (72.7%) were married and received 0–8 years of education (36.4%) or 13 or more years of education (36.4%). All of the participants who screened positive to abuse emigrated from Mainland China.

### 3.2. Perceived Effectiveness of Elder Abuse Interventions

We explored the perceived effectiveness of elder abuse interventions in reducing psychological distress using open-ended questions. Intervention programs identified as most effective to least effective included social support, empowerment, community-based interventions, advocacy, and psychological interventions (Table 2).

Of all the dimensions identified, the majority of responses fell into the category of social support. Specifically, increased peer group support, family support, and community support were viewed as beneficial elements to reduce distress of abused victims. For instance, support group that reduces social isolation and further informs seniors of their rights may be helpful to victims. As one participant described, “If you have a speaker that experienced elder abuse to come in and actually talk to the elders, then elders can connect with someone who understands what they are going through”.

Empowerment was identified as an effective process to help individuals maximize their confidence, skills, and abilities in order to regain control of their lives. Many participants perceived education as the first step to inform victims of their rights. In addition, participants also pointed to increased financial independence of victims as an initial step. Many participants held favorable views toward interventions delivered by community-based social services organizations. Community-based organizations that offer bilingual and bicultural services have been viewed as a safe haven by immigrant older adults and perceived as the only available help resource for immigrants [5–7]. Participants perceived the easy access, prompt response, and flexibility to integrate interventions into existing programs as a plus to community-based intervention module. Advocacy and psychological intervention garnered least favorable views from our data analysis.

### 3.3. Similarities and Differences on Perceptions of Intervention Component between Victims and Nonvictims Groups

In addition, this study investigated the similarities and differences regarding preferences on the intervention components between the victims and nonvictims (Table 3). The majority of responses on preferred intervention programs from victims fell into community-based interventions and empowerment, whereas for nonvictim respondents, empowerment, and social support components received the most favorable views. Psychological interventions, including behavioral interventions and behavioral activations, remained least favorable by both groups.

### 3.4. Perceived Challenges to Each Intervention Component

This study also captured the potential challenges in the design of interventions from the perspectives of older adults (Table 4). A most commonly perceived challenge to advocacy pertains to the fear of limited actions. Participants expressed that advocacy programs would not be effective if the design and implementation only set out to enhance victim self-care without actionable goals. Advocacy could easily be interpreted as “knowledge” or “a type of explanation to abusive situations” and thus may be rendered less effective. Furthermore, participants were aware of the potential implementation barriers to other programs, such as empowerment and psychological interventions. Participants commented that empowerment programs would only be helpful if linking with other elements, such as counseling or peer group support element, that is empowerment does not work; if victims are not provided with direct counseling help (and support), then nothing can be resolved”.

The benefits of psychological interventions to reduce psychological distress of victims remained least evident throughout the discussion. We suspect that the concept of behavioral activation or cognitive behavioral therapy may be foreign to community-dwelling older adults; hence there exists limited understanding on its benefits. Participants expressed the major challenge pertaining to negative perceptions on psychological interventions and behavior interventions. There may be a high level of reluctance and shame in joining such interventions.

Last, many seniors perceive intervention as a way to advocate for victims and their family members that “the community has to do something about it to provide them (victims) the protection and care that they deserve”.

### 3.5. Strategies to Culturally Adapt Elder Abuse Interventions in Chinese Communities

In light of the perceived challenges in the design of interventions, we also solicited ways to adapt existing elder abuse interventions for Chinese seniors in accordance with Chinese cultural and linguistic characteristics (Table 5). Most of the responses fell in the category of improving filial piety practice as a way to improve social support. As one participant claimed, “Parents will feel much better if their children are filial. This is the best measure to help seniors”. The importance of educating and nurturing filial piety values was echoed throughout the discussions.

In addition, our analysis showed that improved peer support by organizing community social events and activities may be a viable option to increase cultural sensitivity of intervention programs. One participant proposed that support group discussions may be integrated with existing cultural programs offered by community centers. Participants also called for more research and educational outreach efforts Health surveys were perceived as a helpful way to equip older adults with necessary health sciences knowledge. In addition, rigorous research contributes to the identification of seniors at high risks of abuse as well as those “who need the most help from us”. Last, increasing religious involvement was recognized as a helpful strategy. Participants linked religious attendance with increased social support, and thus it may be an effective intervention for victims.

## 4. Discussion

In this first exploratory study of perceived effectiveness, challenges, and cultural adaptations of elder abuse interventions among community-dwelling Chinese older adults, our findings suggest that older adults viewed social support, empowerment, and community-based interventions design as most effective to vulnerable victims. The preferences were similar between victim and nonvictims. Strategies to tailor interventions toward Chinese cultural beliefs were also proposed with respect to nurturing filial piety values, familial integration, and improving independence of victims.

This qualitative investigation provides new insights on how minority older adults viewed different abuse intervention components and strategies to overcome intervention barriers. First, our findings provide direction for violence-related intervention services. A multidisciplinary approach that includes strengthening the informal support system including family members, friends, and community gatekeepers as well as facilitating access to formal services building on the health care system warrants exploration in this context.

It has been argued that older adults with fewer psychosocial resources or more psychosocial deficits seem to be more vulnerable to elder abuse, and elder abuse seems particularly detrimental to psychological well-being for victims [34]. Our data suggest that the increased social support from family, friends, and supports is viewed as amongst the most effective interventions in increasing psychosocial resources of victims. This finding resonates with respect to important Chinese traditional values of filial obligations of respect and care from older adults. Despite acculturation challenges brought about by immigration, many traditional values and practices are still entrenched in this community. The utilization of family power in intervention designs warrants further attention.

It is also worth noting that advocacy and psychological interventions received limited appraisal among participants. Advocacy programs aim to enhance abused victims' self-care by helping them to make sense of the situation, identify potential solutions, and achieve the goals they have set [24]. From the limited empirical evidence, the effect of advocacy intervention designed to improve quality of life and mental health of Chinese abused women remained to be seen [14–16]. Similarly, whereas cognitive behavior model remained less favorable, some researches suggest that it can be a viable model in Chinese American population, with a few modifications [35, 36]. Systematic research is needed to explore the effectiveness of the different components of the advocacy and psychological intervention for the various forms of elder abuse.

In addition, given that community-based intervention module is appraised by Chinese older adults in this study, future research is needed to move forward to designing multicomponent elements of interventions suitable for the needs of minority older adults. For instance, studies in tribal communities found that interventions that offer combinations of individual or family counseling, case management, skills training, and behavior management strategies may be more effective than interventions that only provide a peer support group [37]. Measures that provide the opportunity for older adults, caregivers, families, and health professionals to come together to prevent and mitigate elder abuse in a community setting may be a novel option for Chinese older adults.

Furthermore, the results highlight the need to alleviate older adults' fears and shame in disclosing abuse. Our findings reveal that there exist more similarities than differences in the preferences of intervention programs between susceptible victims and nonvictims. Although the finding may be due to a small sample size effect, the violence and mental health stigma may indeed affect older adults' willingness to enroll in intervention programs. Health care providers should consider outreach programs particularly designed to reduce the stigma of violence and caregiver stress among Chinese immigrant families.

Last, conducting participatory research approach allows us to prioritize sensitive cultural context and increase the relevance of our research findings to actionable intervention goals [38, 39]. When culture is continually evolving, the ability to identify factors that are amendable to adaptation to the Chinese population would help ensure that the health intervention is sensitive to the needs and concerns of victims. Our synergetic academic-community collaboration helps raise awareness and understanding of the complexities in elder abuse situations and further empower older adults and their family members.

There are several limitations to this study. First, the data used in this investigation were from a small sample of Chinese older adults. Future studies should consider working with diverse Chinese immigrants in a population-based study design. Second, the sample used was purposively selected from the Chinese community in Chicago, and hence, our results may not be generalizable to other Chinese populations, including suburban groups, Chinese ethnic minority groups, or rural Chinese populations, as they may be subjected to varying degrees of social and economic influence [40]. Systematic examinations of the longitudinal risk and protective factors and of effect size are needed to devise targeted intervention studies. In addition, given the extent of the different types of elder abuse and variation in risk and protective factors and perpetrator characteristics, we did not investigate evidence-based interventions for the specific dyads that may be at particularly high risk for elder abuse. Rigorously designed intervention studies and measures of relevant outcomes of elder abuse are particularly needed.

Nonetheless, this study provides insights on the policy and practice implications for designing and deploying intervention to reduce psychological distress with respect to elder abuse outcome in Chinese aging communities. Cultural relevancy of health interventions is critical in this vulnerable population. Cultural sensitivity in health intervention programs entails more than matching languages or locations preferred by the local community members. Rather, incorporating the cultural, social, and structural contexts in the targeted community contributes to the salience of policy impact [41, 42]. In addition, health care professionals should consider outreach programs particularly designed to increase familial, social, and community support for vulnerable older adults. Collective federal, state, and community efforts are needed to support the culturally appropriate training and education on elder abuse interventions.

## 5. Conclusion

We conclude that, from the perspectives of community-dwelling Chinese older adults, elder abuse interventions hold promise for improving the psychological well-being of victims. Our results further underscore the urgent need of designing culturally and linguistically sensitive evidence-based interventions tailored toward the needs of diverse aging populations.

## Figures and Tables

**Table 1:** Characteristics of the study population by the presence of elder abuse.

Any abuse	Yes (*N* = 11)	No (*N* = 26)
Age group, number (%)		
60–69	5 (45.5)	8 (30.8)
70–79	6 (54.5)	12 (46.2)
80+	0 (0.0)	6 (23.1)
Sex, number (%)		
Men	7 (63.6 )	9 (34.6 )
Women	4 (36.4)	17 ( 65.4)
Education levels, years (%)		
0–8 years	4 (36.4)	12 (46.2)
9–12 years	3 (27.3)	9 (34.6)
13 or more	4 (36.4)	5 (19.2)
Marital status, number (%)		
Married	8 (72.7)	19 (73.1)
Single	1 (9.1)	0
Widowed	2 (18.2)	7 (26.9)
Number of people in the household, number (%)		
1	3 (27.3)	6 (23.1)
2-3	7 (63.6)	14 (53.8)
4 or more	1 (9.1)	5 (19.2 )
Origin, number (%)		
Mainland	11 (100)	25 (96.2 )
Hong Kong	0	0
Taiwan	0	1 (3.8)
Others	0	0
Number of years in the U.S., number (%)		
1–10	3 (27.3)	6 (23.1)
11–20	2 (18.2)	7 (26.9)
21–30	5 (45.5)	10 (38.5)
31 or more	1 (9.1)	3 (11.5)
Language ability, number (%)		
Cantonese	8 (72.2)	17 (65.4)
Mandarin	2 (18.2)	2 (7.7)
English	1 (9.1)	0 (0.0)
Toishanese	0 (0.0)	7 (26.9)

**Table 2:** Perceived effectiveness of elder abuse intervention.

Themes	Subthemes	Representative statements
Social support	Peer group support	“I think you can have increased support from victims themselves, like support group type of things. If you have a speaker that experienced elder abuse to come in and actually talk to the elders, then elders can connect with someone who understands what they are going through. The speaker can share his help-seeking experiences and provide guidance to other peers too.”
	Family support	“As family friends and relatives, we need to be patient and listen to their problems.” “They need family and relatives to lend ears to hear their problems.”
	Community support	“(…) we need to get a support group together in the community centers. Before older adults realize any potential abusive situations, they can go to the support groups and let professionals intervene at the earliest possibility. Then perhaps we can prevent abuse from happening.” “I was thinking that maybe there should be a place for those elders to enjoy the time or to talk about their problems with people there to help.”
	Phone calls and hotlines	“I hope there is a bilingual hotline number to call, if anything comes up.”

Empowerment	Education	“Education is the first step. They (victims) need to have a clear understanding of what elder abuse is so that they know what is going on.” “If everyone conforms to Chinese traditional values to respect seniors, there would be no abuse at all. Therefore, education is important. If we educate everyone, then we can stop abuse.”
	Counseling	“Counseling is important too, so that victims can talk freely about their distress and frustrations.” “I think if a senior is abused, he/she will be very sad and painful. If we don't provide counseling help, we will not be able to relate to that type of turmoil and provide solutions. So I think counseling will be really critical.”
	Enhance financial independence	“We need to improve the financial situations of abused seniors. If they can stand on their own feet financially, it may help them overcome abusive situations and decrease their risks to be victimized.”

Community-based intervention	Interventions delivered through community agencies	“I think the options we have may all be effective. But if I must pick one, I would think community-based intervention would work best in our community, since seniors always turn to community social services agencies for help.” “I think the community-based intervention model would work. If seniors have problems, community organizations can always step in and help.”
	Integrate services to existing social service agencies	“I think we should educate homemakers about elder abuse. On the one hand, they can help us communicate and outreach to seniors. On the other hand, if they encounter any clients who may be victimized by abuse, they can help report the cases. Homemakers should be more aware of these types of issues and advocate for seniors.”

Advocacy	Understand current situation and identify potential solutions	“We need to provide seniors with viable solutions. This could be one of the most important methods. Make sure the seniors are willing to listen. Newspapers and advertisement may be important as ways to advocate for their rights. And provide them with potential solutions and ways out of the abusive relationships.”

Psychological intervention	Behavior activation	“It is important do something positive to alleviate their distress. For instance, I like painting. If I am distressed or frustrated, I would paint. It helps express my feelings and frustrations. These types of changes may help victims as well.”

**Table 3:** Similarities and differences on perceived preferences of intervention components between victims and nonvictims groups.

Among victims	Proportion of responses	Among nonvictims	Proportion of responses
Community-based intervention	31.5%	Empowerment	32.3%
Empowerment	31.5%	Social support	27.9%
Social support	22.2%	Community based intervention	22%
Advocacy	9.3%	Advocacy	8.8%
Psychological	5.5%	Psychological	8.8%

**Table 4:** Perceived challenges in the design of intervention.

Themes	Subthemes	Representative statements
Advocacy	Advocacy will be helpful if linking to actual actions to stop abuse	“I think advocacy is not going to be useful unless you help seniors to take real actions. You need actual changes to stop abuse, not only advocating for the rights of seniors.” “Among all the components, I think advocacy is least useful. Advocacy is only knowledge, a type of explanation to tell people what to do. It is too far reached and actual impact is unclear.”
	The root of the issue is to stop abusers	“We should think about the abuser. Why are they abusing the elderly? Maybe the abusers have some problems or stress management issues; we should also talk to them and see how they feel.”

Empowerment	Empowerment will be helpful if linked with counseling	“Empowering seniors is an added value to the intervention. It does not help solve the real problem.” “Empowerment does not work. If victims were not provided with counseling …(and support), nothing can be resolved. Victims will still feel lonely and frustrated.”

Psychological intervention	The concept may be foreign to community seniors and hence decrease the chance of using this intervention	“Some people don't even want your help, they would be like, go away, I don't need it, it's going to make my life worse for me…that's what older people might think...Are they going to accept that? If they go to therapy, they may say that they don't think they have any problems. Older people have that attitude. So how are you going to persuade them to go to therapy and change any types of behaviors?”

**Table 5:** Strategies to culturally adapt elder abuse interventions in the chinese communities.

Themes	Subthemes	Representative statements
Improve social support	Improve and nurture filial piety	“Parents will feel much better if their children are filial. This is the best way to help seniors. Much better than improving other types of social support. So we need to educate our children and grandchildren and try to avoid family conflicts.” “Abusive situations can be much improved upon if children are filial. If they respect seniors, they will feel much better. Wouldn't you say that this is the best way to improve the mental health of victims?”
	Improve peer support	“We can organize more social events and activities for older adults. I think Chinese seniors are often lonely. Especially those who may encounter problems and conflicts at home. If we can get them to go to those support groups, it will help ease their minds.” “Seniors need to make more friends, especially with those who are currently undergoing similar issues. We need to get Chinese victims all together, like having them go to the same group discussion in community centers after some sort of painting classes or tai-chi classes.”

Provide financial independence	Enhance victims' financial independence	“The U.S. government should be attentive of the social welfare needs of immigrant older adults. I think if abused seniors have more financial means, or financially more stable, they may not need to depend on the abusers at all, which will help improve their independence.”

Research	Provide education through research and workshops	“We need to organize more educational workshops and group activities. It will be helpful if seniors are educated and informed of the benefits to interventions.” “We need more research and health surveys that gather comprehensive health information of seniors. We will be able to document the health and well-being of seniors this way and reach out to those who need the most help from us.”

Religion	Provide victims with religious support	“I believe we can try to help them by increasing their religion involvement. If they have higher religious attendance, they may be able to get more social support as well.”
